# Effect of Metformin on Cancer Risk and Treatment Outcome of Prostate Cancer: A Meta-Analysis of Epidemiological Observational Studies

**DOI:** 10.1371/journal.pone.0116327

**Published:** 2014-12-29

**Authors:** Hongliang Yu, Li Yin, Xuesong Jiang, Xiujin Sun, Jing Wu, Hao Tian, Xianshu Gao, Xia He

**Affiliations:** 1 Department of Radiation Oncology, Jiangsu Cancer Hospital & Jiangsu Institute of Cancer Research, the Affiliated Cancer Hospital of Nanjing Medical University, Nanjing, Jiangsu Province, P. R. China; 2 Department of Radiation Oncology, Peking University First Hospital, Peking University, Beijing, P. R. China; IPO, Inst Port Oncology, Portugal

## Abstract

**Background:**

Laboratory studies have shown the anti-tumor effect of metformin on prostate cancer. However, recent epidemiological studies have yielded inconclusive results.

**Methods:**

We searched PubMed database from the inception to May 30 2014 for studies which assessed the effect of metformin use on cancer risk of prostate cancer, biochemical recurrence (BCR) and all-cause mortality of patients with prostate cancer. The pooled results and 95% confidence intervals (CIs) were estimated by random-effect model.

**Results:**

Twenty-one studies were eligible according to the inclusion criteria. Based on the pooled results of available observational studies, metformin use was significantly associated with a decreased cancer risk (14 datasets, 963991 male subjects, odds ratio: 0.91, 95% CI: 0.85–0.97) and BCR (6 datasets, 2953 patients, hazard ratio: 0.81, 95% CI: 0.68–0.98) of prostate cancer. However, the association of metformin use with all-cause mortality of patients with prostate cancer was not significant (5 datasets, 9241 patients, hazard ratio: 0.86, 95% CI: 0.64–1.14).

**Conclusion:**

Results suggest that metformin use appears to be associated with a significant reduction in the cancer risk and BCR of prostate cancer, but not in all-cause mortality of patients with prostate cancer.

## Introduction

Prostate cancer is the most common male malignancy in the Western world, and the incidence in Asian countries has been increasing significantly in past decades [1]; therefore, methods for preventing and curing this malignancy are urgently needed.

Metformin is the most widely used oral hypoglycemic agent in type 2 diabetes, and it has a favorable toxicity profile and extremely low cost. Its primary action is the inhibition of hepatic glucose production through an LKB1/AMPK–mediated mechanism, and it also improves insulin sensitivity in peripheral tissues [2]. Recently, metformin has gained increasing interest in the medical community for its potential antitumorigenic effects [3]. Epidemiological studies have demonstrated that metformin can reduce the risk of breast, colon, pancreatic, and liver cancers and might even improve cancer prognosis [4]. Preclinical studies also have shown the beneficial effects of metformin on prostate cancer cells, as it can inhibit cell proliferation and induce the apoptosis of prostate cancer cell lines in vitro and in vivo [5]. However, epidemiologic studies have yielded inconsistent results; some of these works have shown that metformin indeed decreased the cancer incidence and promoted an improved cancer prognosis [6]–[10], while other studies found no such associations [11]–[13]. With these premises, we performed a systematic review and meta-analysis of the currently available studies to comprehensively explore the effects of metformin on both cancer prevention and the treatment outcomes of prostate cancer, specifically, its preventative qualities in regard to cancer risk and on its BCR as well as the all-cause mortality for the treatment outcomes of prostate cancer.

## Methods

### Data sources and research

Relevant studies were identified by searching the PubMed database (www.ncbi.nlm.nih.gov/pubmed), within the time frame from its inception to May 30, 2014. We limited the searches to studies in humans and published in English-language journals. We used relevant text words and medical subject headings that included “hypoglycemic agents” or “metformin” or “biguanides” in combination with “prostate” or “prostatic” and “cancer” or “neoplasm” or “carcinoma”. The reference lists of the identified articles were manually scanned to identify any other relevant studies. The ClinicalTrials.gov website was also searched for randomized trials that were registered as completed but not yet published. In addition, the references for the reviews and meta-analyses covered on this issue were also scrutinized to identify additional relevant publications [14]–[16].

### Inclusion and exclusion criteria

The inclusion and exclusion criteria for this meta-analysis were as follows: the work (1) should be a published observational study or randomized clinical trial evaluating the impact of exposure to metformin compared with a comparison group on the cancer risk and/or treatment outcomes of prostate cancer; (2) must have reported relative risks (RR), hazards ratios (HR) or odds ratios (OR) and 95% confidence intervals to estimate the effect of metformin on the cancer risk or treatment outcomes (BCR or all-cause mortality) of patients with prostate cancer; (3) must have clear information on the adjustments for confounding factors; and (4) must be an independent study to avoid assigning a double weight to estimates derived from the same study published twice or more.

### Data extraction and study quality assessment

The results of the search strategy and the identified eligible studies were reviewed by two authors (Hongliang Yu and Xiujin Sun) independently. For each eligible study, we extracted the data using a standardized data-collection form, including details on the authors, study country, publication year, study design, study period, total number of male subjects and the number of cases of prostate cancer, comparison groups, effect estimates, adjustments or stratification variables and study quality. Discrepancies in the data extraction between the two reviewers were resolved by discussion.

The study quality was assessed by applying a 9-star system on the basis of the Newcastle-Ottawa Scale (NOS) [17], and a study with ≥7 awarded stars was defined as a high-quality study.

### Statistical analysis

We pooled the effect estimates from the individual studies using a random effects model, which considered both within- and between-study variations, yielding more conservative results than the fixed-effect model [18]. For the cancer risk analysis, the OR was used as the common measure of association across studies, and the RR and HR were directly considered as the OR for the cancer incidence was relatively low [19]. For the BCR and all-cause mortality analysis, the HR was used as the common measure. Heterogeneity was evaluated using the I^2^ test, which represents the percentage of total variation across studies that is attributable to heterogeneity rather than to chance [20]. We also conducted subgroup analyses according to the study location, study design, comparison group and study population to assess the potential modification effects of these variables on the risk of prostate cancer. Additionally, a sensitivity analysis to investigate the influence of a single study on the overall risk estimate by omitting one study in turn was performed for all of the analyses. The potential publication bias was assessed by Begg's funnel plots and Egger's regression test [21]. A p value <0.05 was considered to be statistically significant, except where otherwise specified. All statistical analyses were performed with STATA version 12.0 (StataCorp).

## Results

### Literature search

**Figure 1:**
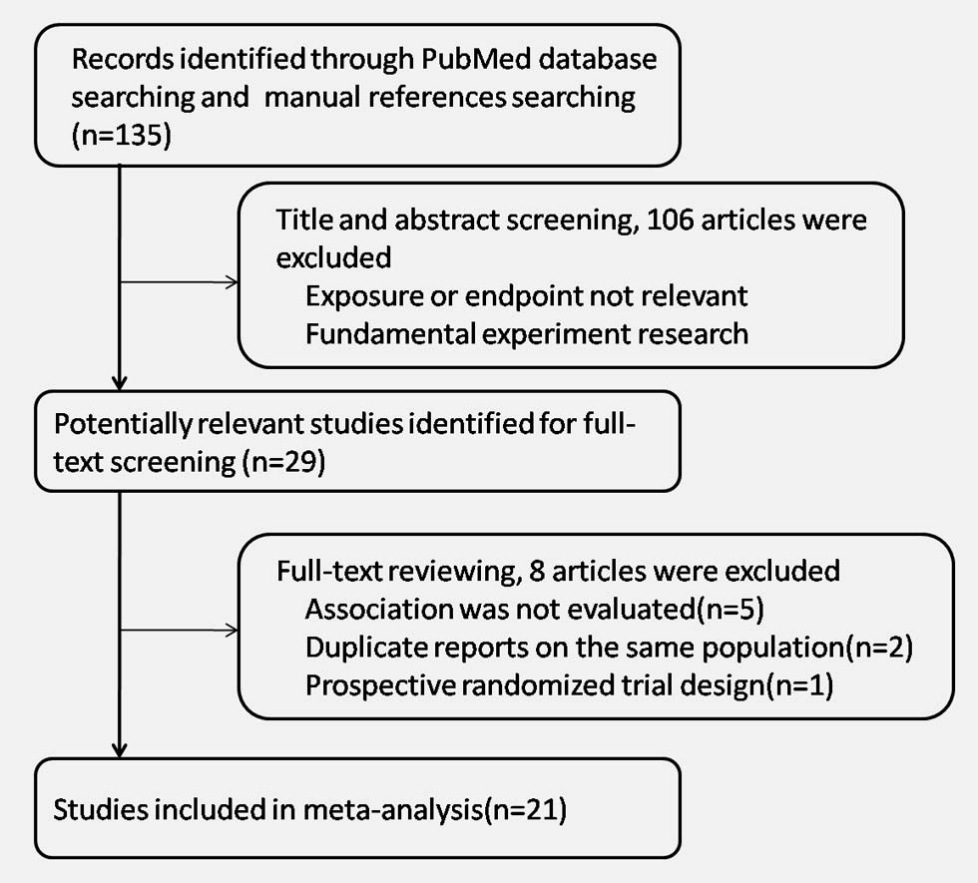
Flow chart of the study selection.

We initially identified 135 potentially eligible studies by title and abstract screening, but most of them were excluded because the exposure or endpoint was not relevant to our analysis or they were fundamentally biochemical experimental research. After assessing the fulltext of the 29 potentially relevant studies, we identified 21 eligible studies [6]–[13], [22]–[34] for analyses. The primary reasons for exclusion were as follows: 5 studies did not specify the association between metformin and the cancer risk or treatment outcome of prostate cancer; the other 2 studies [35], [36] were duplicate reports of one included study [11] on the same populations; 1 study [37] was reported to be a prospective randomized trial design, but its initial intent was not to explore the relationship between metformin use and the risk of prostate cancer; it also did not clearly state the adjustments for confounding factors. As a result, all of the studies included in this study were retrospective in design. A flow chart showing the study selection process is presented in Fig. 1.

### Study characteristics

The characteristics of the included studies are presented in Tables 1, 2, and 3. Of the included studies, nearly all of them were conducted in Western countries, 15 in North America and 6 in Europe; only 2 of the studies were conducted in East Asia in Taiwan. Of the 12 observational studies conducted to explore the association between metformin use and the risk of prostate cancer, 7 were retrospective cohort in design and 5 were case-control in design. The total number of all of the included male subjects in the cancer risk analysis was 963991. For the analyses of the effect of metformin on BCR and the all-cause mortality of prostate cancer, all of the studies were retrospective cohorts in design, and the total number of prostate cancer cases included was 2953 and 9241, respectively. We also observed that all of the studies were conducted between 2008 and 2014, which appears to reflect the currently rising interest of the medical community in the potential benefits of metformin use for prostate cancer.

**Table 1:** Characteristics of the included studies for the risk of prostate cancer analysis.

Author publication year	Country/region	Study design	Study period(years)	Male subjects	PCa cases	Treatment comparison	Measure of outcome	Adjustments*	Study quality score
Currie et al., 2009	UK	retrospective cohort study	2.4 (2000–2003)	32261	301	metformin vs Sulfonylureas; metformin vs Insulin-based therapies	aHR	1–3	7
Ferrara et al., 2011	USA	retrospective cohort study	8 (1997–2005)	134864	2105	metformin vs pioglitazone	aHR	1,2,5,9,13,15	5
Azoulay et al., 2011	Canada	nested case–control study	21(1988–2009)	8098	739	Metformin vs other hypoglycemic agent	aRR	2,3,5,6,9,12,16	7
Murtola et al., 2008	Finland	Case-Control Study	7(1995–2002)	49446	24723	Metformin user vs nonuser	aOR	1,4,16	6
Wright et al.,2009	USA	Case-Control Study	3(2002–2005)	1943	1001	Metformin user vs nonuser	aOR	1,5–8,16	6
Hsieh et al.,2012	Taiwan	retrospective cohort study	8(2000–2008)	5680	84	metformin vs Insulin; metformin vs Sulfonylureas	aOR	1	6
Onitilo et al.,2013	USA	retrospective cohort study	14(1995–2009)	4956	237	Metformin vs other hypoglycemic agent	aHR	1,2,4,6,10,14,15	6
Ruiter et al.,2012	Netherlands	retrospective cohort study	10(1998–2008)	40131	236	metfromin vs Sulfonylurea derivatives	aHR	1,5,10	8
Morden et al.,2011	USA	Retrospective cohort study	5(2003–2008)	25660	2072	metformin vs insulin	aHR	1,2,6,10,13,15	5
Margel et al.,2013	Canada	nested case–control study	15(1994–2009)	31836	5306	Metformin vs other hypoglycemic agent	aOR	4–6,10,15,16	7
Tseng,2011	Taiwan	retrospective cohort study	2(2003–2005)	494630	889	Metformin user vs nonuser	aHR	1,4–6,10,15,16	5
Preston et al.,2014	Denmark	nested case–control study	22(1989–2011)	134486	12226	Metformin user vs nonuser	aOR	10,16	8

*Adjustments: 1.Age, 2.smoking status, 3.prior cancer diagnosis, 4.place of residency, 5.use of other diabetes medications, 6.BMI, 7.PSA value, 8.family history of prostate cancer, 9.HbA1c, 10.Comorbidities, 11.Townsend index of deprivation, 12.excessive alcohol use, 13.race/ethnicity, 14.year of diagnosis, 15.socioeconomic status, 16.other drug use, 17.ADT treatment, 18.T stage, 19.Gleason score, 20.primary treatment with radiation or surgery, 21.surgical margin, 22.lymph node metastasis, 23. extracapsular extension, 24. seminal vesicle invasion.

**Table 2:** Characteristics of the included studies for the BCR of prostate cancer analysis.

Author publication year	Country/region	Study design	Study period(years)	PCa cases	Patients undergo BCR	Treatment comparison	Measure of outcome	Adjustments*	Study quality score
Allott et al.,2013	USA	retrospective cohort study	22(1988–2010)	371	134	prostatectomy+metformin vs prostatectomy without metformin	aHR	1,6,7,13,18,19,21,23,24	8
Kaushik et.al.,2013	USA	retrospective cohort study	13(1997–2010)	885	203	prostatectomy+metformin vs prostatectomy without metformin use	aHR	1,6,7,16,18,19,21	8
Patel et al.,2010	USA	retrospective cohort study	19(1990–2009)	210	79	prostatectomy+metformin vs prostatectomy without metformin use	aHR	1,7,18,21,22	8
Rieken et. al.,2013	USA and Europe	retrospective cohort study	11(2000–2011)	664	173	prostatectomy+metformin vs prostatectomy without metformin use	aHR	1,7,19,21–23,24	7
Spratt et al.,2013	USA	retrospective cohort study	16(1992–2008)	319	79	radiotherapy+metformin vs radiotherapy without metformin	aHR	1,7,17–19	8
Zannella et al.,2013	Canada	retrospective cohort study	16(1996–2012)	504	165	radiotherapy+metformin vs radiotherapy without metformin	aHR	1,7,16–19	7

* Adjustments: 1.Age, 2.smoking status, 3.prior cancer diagnosis, 4.place of residency, 5.use of other diabetes medications, 6.BMI, 7.PSA value, 8.family history of prostate cancer, 9.HbA1c, 10.Comorbidities, 11.Townsend index of deprivation, 12.excessive alcohol use, 13.race/ethnicity, 14.year of diagnosis, 15.socioeconomic status, 16.other drug use, 17.ADT treatment, 18.T stage, 19.Gleason score, 20.primary treatment with radiation or surgery, 21.surgical margin, 22.lymph node metastasis, 23. extracapsular extension, 24. seminal vesicle invasion.

**Table 3:** Characteristics of the included studies for the all-cause mortality of the prostate cancer analysis.

Author publication year	Country/region	Study desingn	Study period(years)	PCa cases	Patients death	Treatment comparison	Measure of outcome	Adjustments*	Study quality score
Spratt et al.,2013	USA	retrospective cohort study	16(1992–2008)	2901	199	radiotherapy+metformin vs radiotherapy without metformin	aHR	1,7,17–19	8
He et.al.,2011	USA	retrospective cohort study	9(1999–2008)	233	/	ever vs never use of metformin	aHR	1,6,13,18,19	8
Margel et al.,2013	Canada	retrospective cohort study	12(1997–2009)	3837	1343	ever vs never use of metformin	aHR	1,10,14–18,20	9
Kaushik et.al.,2013	USA	retrospective cohort study	13(1997–2010)	885	94	prostatectomy+metformin vs prostatectomy without metformin	aHR	1,6,7,16,18,19,21	8
Currie et al., 2012	UK	retrospective cohort study	19(1990–2009)	1385	465	ever vs never use of metformin	aHR	1,2,10,11,14	8

* Adjustments: 1.Age, 2.smoking status, 3.prior cancer diagnosis, 4.place of residency, 5.use of other diabetes medications, 6.BMI, 7.PSA value, 8.family history of prostate cancer, 9.HbA1c, 10.Comorbidities, 11.Townsend index of deprivation, 12.excessive alcohol use, 13.race/ethnicity, 14.year of diagnosis, 15.socioeconomic status, 16.other drug use, 17.ADT treatment, 18.T stage, 19.Gleason score, 20.primary treatment with radiation or surgery, 21.surgical margin, 22.lymph node metastasis, 23. extracapsular extension, 24. seminal vesicle invasion.

The quality score of the included studies ranged from five to nine stars on the scale, and the median score of the included studies for the cancer risk, BCR and all-cause mortality analysis was 6, 8 and 8, respectively. The prevalence of a study quality lower than 7 in the cancer risk analysis may be because many of the included studies were not conducted not directly foucused on exploring the association between metformin use and the risk of prostate cancer [7], [23], [24], [26], [32].

### Primary analysis

**Figure 2:**
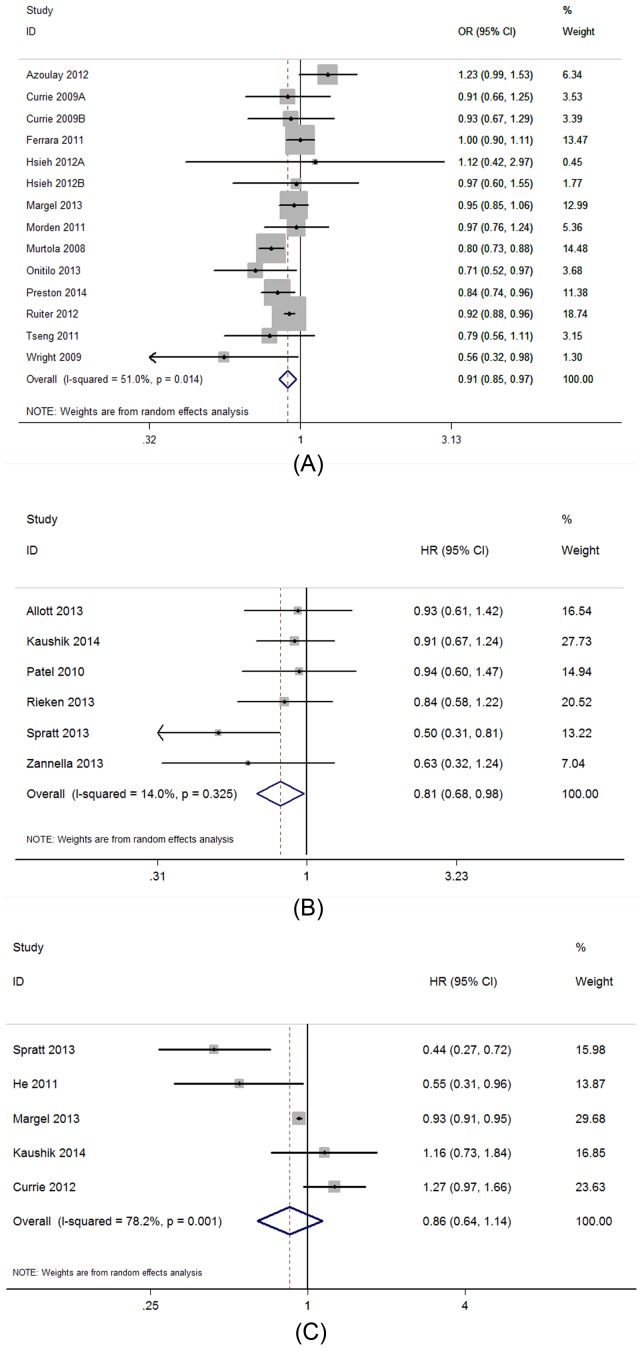
Forest plot of the primary results. Random-effects meta-analyses of observational studies that examined metformin use and (A) prostate cancer risk, (B) the BCR of prostate cancer after treatment and (C) the all-cause mortality of prostate cancer. Squares indicate the study-specific relative risks (the size of the square reflects the study-specific statistical weight); horizontal lines indicate 95% confidence intervals; diamonds indicate a summary OR estimate with its corresponding 95% confidence interval.

The primary results of the meta-analysis are presented in Fig. 2. A forest plot graph of the association between metformin use and the risk of prostate cancer is shown in Fig. 2A, while Fig. 2B and 2C show the effect of metformin use on the BCR and all-cause mortality of patients with prostate cancer after treatment, respectively.

As shown in Fig. 2A, metformin use showed a statistically significant beneficial effect on the risk of prostate cancer, with a summary OR of 0.91 (95% CI: 0.85–0.97). Moderate heterogeneity was found across the studies (*I^2^* = 51%, *p* = 0.01).

The adjusted HR of each study and the summary HR for the effects of metformin use on the BCR of prostate cancer are shown in Fig. 2B. The summary HR was 0.81 (95% CI: 0.68–0.98), which demonstrated a statistically significant beneficial effect of metformin use on the BCR of prostate cancer. Little evidence of heterogeneity was found across the studies (*I^2^* = 14%, *p* = 0.33).

We also studied the effect of metformin use on the all-cause mortality, which is a representive of the overall survival of patients with prostate cancer. As shown in Fig. 2C, in the included individual study showing converse results, the analysis failed to show a significant beneficial effect of metformin on the all-cause mortality of prostate cancer, with a summary HR of 0.86 (95% CI:0.64–1.14). Heterogeneity was found to be statistically significant across the studies (*I^2^* = 78%, *p*<0.01).

### Subgroup and sensitivity analyses

To explore the potential source of the heterogeneity across studies, we performed a subgroup study. Because there were differences in the study locations, study design and comparison groups within the included studies that could markedly modify the results of the included studies, we investigated the influence of these subgroups. The subgroup study for the cancer risk analysis is shown in Table 4. The subgroup study showed that little evidence of between-subgroup heterogeneity was observed in the studies subgrouped by study location and study design, whereas significant between-subgroup heterogeneity was observed between the studies subgrouped by the comparison group design (*I^2^* = 92%, *p*<0.01). Because of the limited number of included studies for the BCR and all-cause mortality analyses, subgroup studies were not performed for these two analyses.

**Table 4:** Subgroup analysis of the effect of metformin use on the cancer risk of prostate cancer.

Group	No. of data sets	OR(95% CI)	Heterogeneity within subgroups		Heterogeneity between subgroups	
			*I^2^*	p	*I^2^*	*p*
Total	13	0.92(0.85–0.99)	52%	0.02	−	
Study location					0	0.45
North America	6	0.95(0.84–1.08)	60%	0.05	-	
Europe	5	0.87(0.80–0.94)	51%	0.08	-	
East Asia	3	0.87(0.66–1.13)	0	0.69	-	
Study design					0	0.66
Retrospective cohort study	9	0.93(0.89–0.96)	0	0.64	-	
Case-control study	5	0.91(0.75–1.11)	78%	<0.01	-	
Comparison group					92%	<0.01
Metformin vs other hypoglycemic agents	10	0.95(0.90–1.01)	22%	0.24	-	
Metformin user vs nonuser	4	0.81(0.75–0.87)	0	0.56	-	

Sensitivity analyses were conducted to verify the effect of each study on the overall estimate by omitting one study at a time and calculating the combined results for the remaining studies. The sensitivity analyses are shown in S1 Fig. The results of the sensitivity study for the cancer risk analysis showed good consistency, and omitting any one of the included studies did not significantly affect the combined estimate, with a fairly narrow range of results, from 0.89 (95% CI: 0.84–0.95) to 0.93 (95% CI: 0.87–0.99), indicating that the pooled estimate of our analysis was statistically robust. The sensitivity study for the BCR analysis showed that the study by Spratt et al. [10] affected the summary estimate foremost, and omitting this study yielded a result of 0.88 (95% CI: 0.73–1.05), whereas omitting the remaining studies one at a time yielded fairly consistent results, ranging from 0.78 (95% CI: 0.63–0.98) to 0.83 (95% CI: 0.68–1.01). The sensitivity study for the all-cause mortality analysis showed a poor consistency of the results, with a wide range, from 0.75 (95% CI: 0.51–1.10) to 0.98 (95% CI: 0.77–1.26).

### Publication bias

The Begg's funnel plots for the three meta-analyses did not demonstrate any substantial asymmetry, which was shown in S2 Fig. Egger's regression test also indicated little evidence of publication bias, with p value of 0.85, 0.45 and 0.74, respectively, for the cancer risk, BCR and all-cause mortality analyses.

## Discussion

Metformin has recently garnered increasing interest from the medical community for its potential beneficial effects on prostate cancer. Much of the work dedicated to this issue has been performed in the past 5 years and has yielded inconsistent results. This study is the first comprehensive meta-analysis and systematic review of the available studies focused on the effects of metformin both on the prevention and treatment of prostate cancer.

The combined 12 epidemiologic studies and 14 datasets showed that metformin use lowered the cancer risk by 9%, with a summary OR of 0.91 (95% CI: 0.85–0.97, *p*<0.01). The BCR can indicate disease progression years before clinical signs or symptoms develop and most likely implies the failure of prostate cancer treatment [38]. Therefore, the association between metformin use and the BCR of prostate cancer was also studied in this study. The meta-analysis of the 6 available retrospective cohort studies showed that metformin use significantly reduced the risk of a BCR of prostate cancer after treatments, with a summary HR of 0.81 (95% CI: 0.68–0.98, *p* = 0.03). Several mechanisms may be involved in the beneficial effect of metformin for prostate cancer. Studies have revealed that metformin may activate the LKB1/AMPK signal pathway, inhibit protein synthesis, and induce cell cycle arrest and/or cell apoptosis, as well as eradicate cancer stem cells [3], thereby potentially reducing the cancer risk and BCR after the treatment of prostate cancer.

Prostate cancer is a disease with a slow progression, and patients may die with this disease but not of this disease [38]. Metformin can affect the metabolism and inner environment of the human body in multiple ways. How metformin affects the all-cause mortality of prostate cancer is also a concern of this study. Due to the limited number of available studies and the markedly converse study results, the summary result of 5 retrospective cohort studies failed to show a significant beneficial effect for metformin on the all-cause mortality of prostate cancer, with a summary HR of 0.86 (95% CI: 0.64–1.14, *p* = 0.29).

Heterogeneity is often a concern of a meta-analysis. We found a moderate heterogeneity (*I^2^* = 51%, p = 0.01) existing in the analysis of the association of metformin use and the cancer risk of prostate cancer. To investigate the validity of our result and the source of the heterogeneity, we performed sensitivity and subgroup studies. The sensitivity study yielded consistent results; the ORs after omitting one study at a time were all statistically significant and similar to one another, indicating that our results were statistically robust. The subgroup study showed that the differences in the comparison group design and details such as metformin vs. other hypoglycemic agents in patients with diabetes or users vs. nonusers of metformin in the general population significantly contribute to the overall heterogeneity. The potential rationale for this may be the difference between the nature of the two comparator groups. As non-user of metformin group involes both patients with other hypoglycemic agents and population without any diabetic drugs. Besides, some diabetic medications other than metformin may also affect the incidence of prostate cancer [6], [23]. lastly, the association between diabetes, other than hypoglycemic agent use, and risk of prostate cancer could still not be ruled out as a cofounding fact. Nevertheless, our findings support Preston's study [34], metformin users had a more pronounced reduction in cancer risk compared with users of other diabetic medications or no diabetic medications. In our study, substantial heterogeneity was also found in the all-cause mortality analysis. Many reasons may contribute to it. Firstly, only a few studies were available for evaluating the association of metformin use and all-cause mortality of patients with prostate cancer. The study design in these studies were with obvious differences. For example, the study by Kaushik et al [13] investigated patients with prostate cancer treated with surgery, while patients in the study by Spratt et al [10] were treated with radiotherapy. In the study by Margel et al [25], they used cumulative use of metformin as the exposure and evaluated the dose-response effect of metfromin on the results, yielded significant benefit effect of metformin use on the all-cause mortality. While Currie et al [29] defined exposure as ever exposed to metformin immediately before and after cancer diagnosis, yielded an opposite result. Secondly, all-cause mortality may be influenced by many facets of facts, such as characteristics of study population, the stage and treatments of cancer, and the comorbidity of the included patients. All these may induce heterogeneity in the all-cause mortality analysis. For the meta-analysis of the association between metformin use and the BCR after treatment of prostate cancer, little evidence of heterogeneity was found (*I^2^* = 14%, *p* = 0.33). Because of the limited number of available studies for the BCR and all-cause mortality analyses, a subgroup study was not performed for these two analyses.

Several previous studies have evaluated the effect of metformin use on the risk of cancers in patients with type 2 diabetes [14]–[16], [39]. However, this study is the first study which specifically focused on exploring the association between metformin and the risk of prostate cancer. Moreover, our study identified 12 studies with 14 data sets and included 963991 male subjects, a much larger population than any existing analysis [15], [16]. As a consequence, our results gained much stronger statistical power. Additionally, our results are consistent with previous studies suggesting a protective role of metformin for the prevention of prostate cancer [15]. Nevertheless, we still suggest that further studies should be undertaken to confirm or refute the results of our analyses.

In addition to the cancer risk, this study also studied the association between metformin use and treatment outcomes, which includes the BCR and all-cause mortality of prostate cancer. To the best of our knowledge, it is the first study focused on this issue, which may be partially attributable to the fact that the available literature on this topic is limited and novel; all of the studies were published in the past five years and more than half of the included studies were published later than 2012.

The generalization of our findings is mainly hampered by the retrospective nature of the included studies, which were prone to selection or information bias and could lead to an overestimate of the effect [40]. Although we scrutinized carefully the available prospective trial studies on this issue, we found only a few, which were not initially designed to explore the proposed questions of our interest [37]. In addition, some of the included studies [7], [22], [26] were based on medical records or insurance data that were not primarily designed to assess the effect of metformin use on prostate cancer, and the details on the dose, duration, variation over time in the process of treatment as well as full information on the potential confounders were incomplete; as a consequence, the dose-response relationship was not clarified in this meta-analysis.

In summary, based on the available observational studies, our analyses demonstrated metformin use was associated with 9% lower risk of prostate cancer, and with an 18% reduction in the BCR after the treatment of prostate cancer for metformin use. However, we failed to achieve a statistically significant association between metformin use and the all-cause mortality of patients with prostate cancer. Although any conclusions without a large-scale prospective randomized study should be cautious, considering the high prevalence of prostate cancer in Western countries and its rising incidence in the world [1], as long as the low cost and favorable toxicity of metformin, the applicability of metformin use as recommendation for prevention or treatment of prostate cancer may suit multiple populations, in people both with or without type 2 diabetes, and in both developed or developing countries. We expect further experimental investigations will be conducted to clarify the effect of metformin on prostate cancer. In fact, pioneers have already begun to act [41], [42].

## Supporting Information

S1 Fig **Sensitivity analysis for the primary results.** Sensitivity analyses for the effect of metformin use on (A) prostate cancer risk, (B) the BCR of prostate cancer and (C) the all-cause mortality of prostate cancer. The analysis was conducted by omitting each study in turn. Meta-analysis random-effects estimates were used. The two ends of the dotted lines represent the 95% CI.
(TIF)

S2 Fig **Begger's funnel plot of the publication bias for the primary results.** Begger's funnel plot of the publication bias for (A) the prostate cancer risk analysis, (B) the BCR analysis and (C) the all-cause mortality analysis. Each dot represents a separate study for the indicated association.
(TIF)

S1 PRISMA Checklist **The PRISMA checklist for this meta-analysis.**
(DOC)
