# Supplementary figures and images for: Effect of Metformin on Cancer Risk and Treatment Outcome of Prostate Cancer: A Meta-Analysis of Epidemiological Observational Studies
Source: PLoS One. 2014 Dec 29;9(12):e116327. doi: 10.1371/journal.pone.0116327 (PMC4278883; doi:10.1371/journal.pone.0116327)

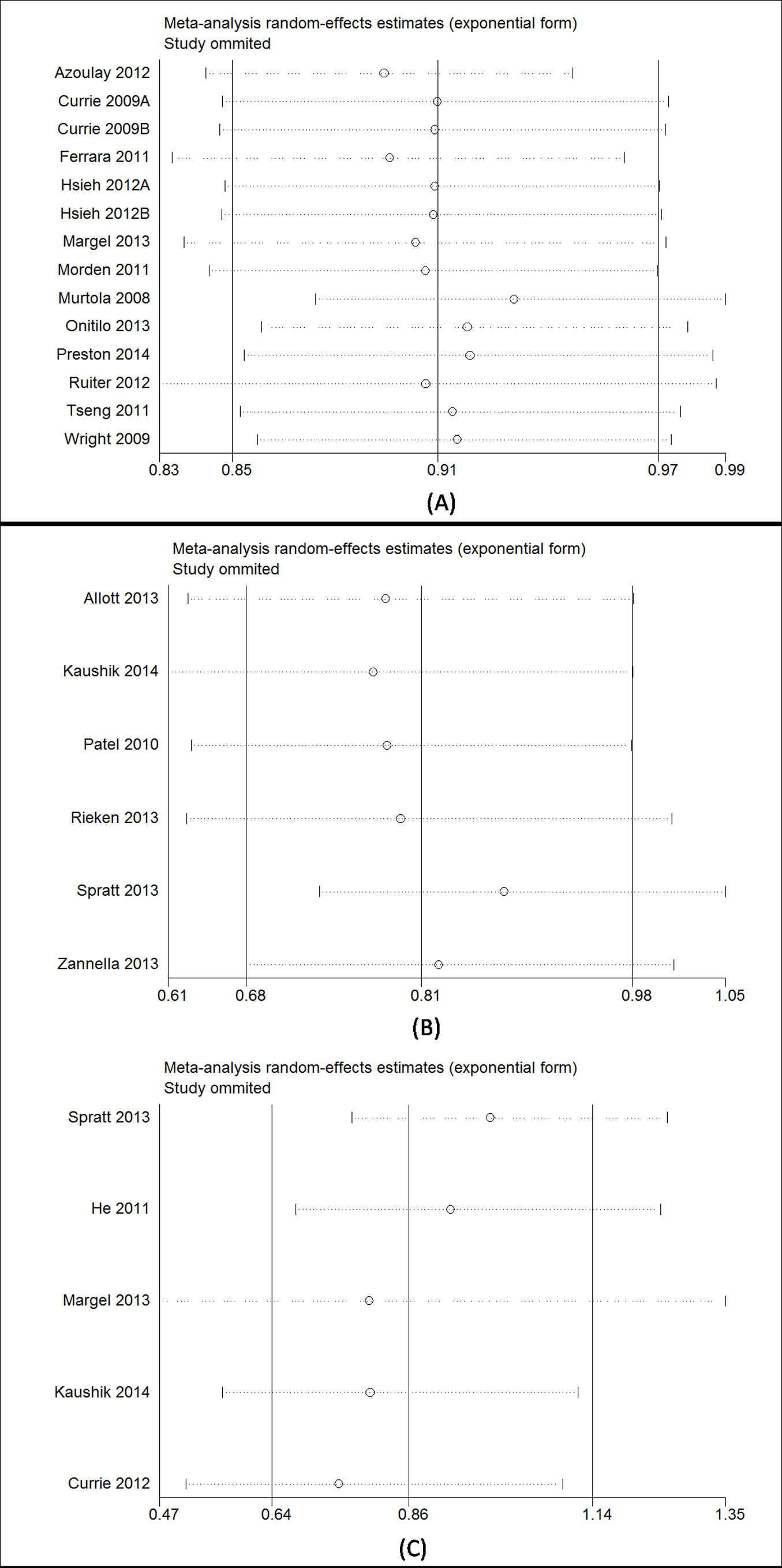

Supplement: S1 Fig — Sensitivity analysis for the primary results. Sensitivity analyses for the effect of metformin use on (A) prostate cancer risk, (B) the BCR of prostate cancer and (C) the all-cause mortality of prostate cancer. The analysis was conducted by omitting each study in turn. Meta-analysis random-effects estimates were used. The two ends of the dotted lines represent the 95% CI. (TIF) [file pone.0116327.s001.tif]

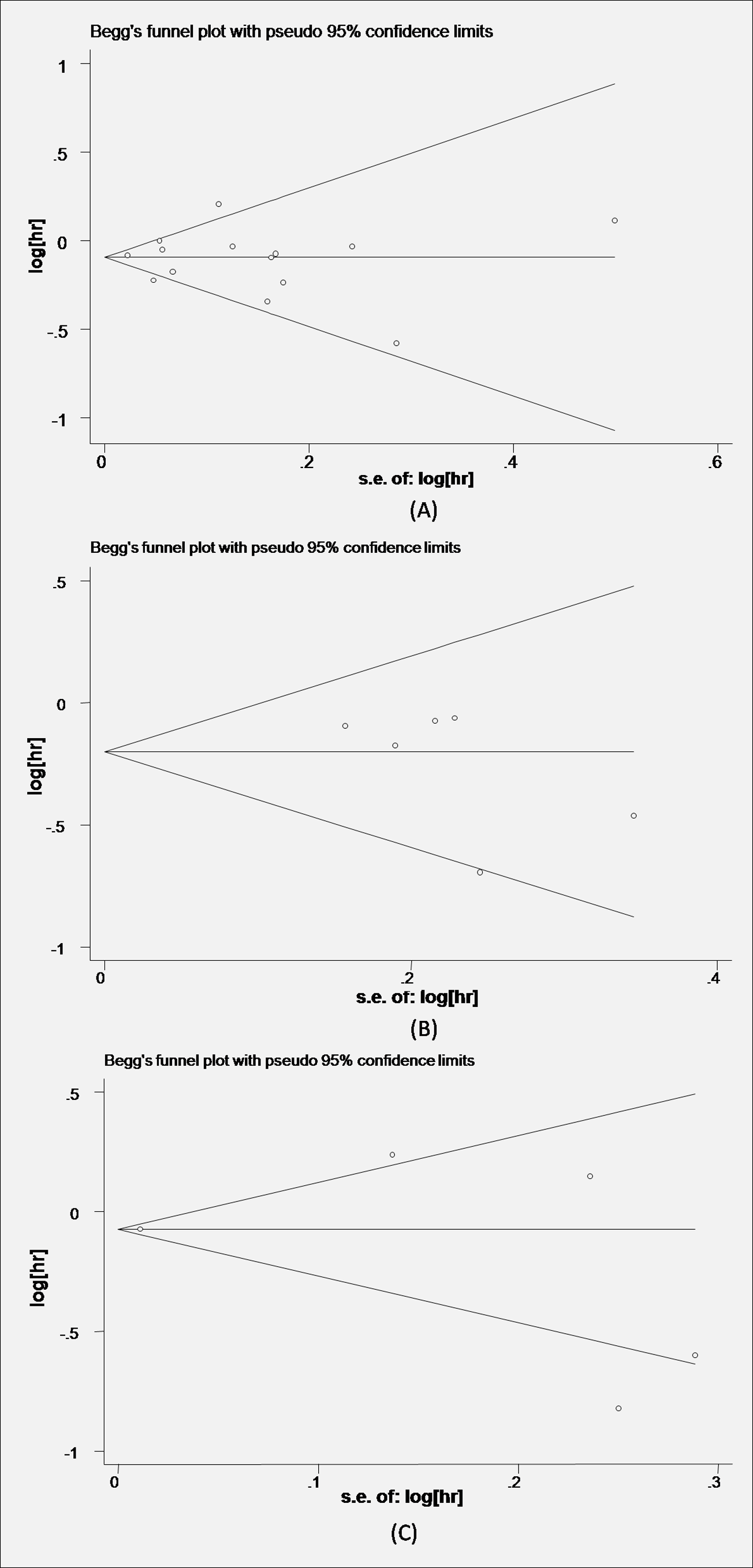

Supplement: S2 Fig — Begger's funnel plot of the publication bias for the primary results. Begger's funnel plot of the publication bias for (A) the prostate cancer risk analysis, (B) the BCR analysis and (C) the all-cause mortality analysis. Each dot represents a separate study for the indicated association. (TIF) [file pone.0116327.s002.tif]
